# Luteolin Enhances Choroid Plexus 5-MTHF Brain Transport to Promote Hippocampal Neurogenesis in LOD Rats

**DOI:** 10.3389/fphar.2022.826568

**Published:** 2022-03-25

**Authors:** Hui-Zhen Li, Kai-Ge Liu, Ning-Xi Zeng, Xiao-Feng Wu, Wen-Jun Lu, Han-Fang Xu, Can Yan, Li-Li Wu

**Affiliations:** Research Center for Basic Integrative Medicine, Guangzhou University of Chinese Medicine, Guangzhou, China

**Keywords:** late-onset depression, luteolin, choroid plexus folate transporters, hippocampal neural stem cells, 5-methyltetrahydrofolate

## Abstract

Folates, provided by food, are commonly used antidepressant synergists in late-onset depression (LOD). However, increased intake of folic acid in the elderly population might lead to the accumulation of unmetabolized folic acid in the systemic circulation, leading to enhanced deterioration of the central nervous system function. In addition, folates cannot access the brain directly because of the blood–brain barrier. Choroid plexus (CP) 5-methyltetrahydrofolate (5-MTHF) brain transport plays a critical role in regulating the cerebrospinal fluid (CSF) 5-MTHF content. Luteolin is a natural flavonoid that has antidepressant effects and is involved in the anti-folate resistance pathway. It remains unclear whether the antidepressant effects of luteolin are associated with the CP 5-MTHF brain transport. In this study, 20–21-month-old Wistar rats were exposed to the chronic unpredictable mild stress (CUMS) protocol for 6 consecutive weeks to explore the long-term effects of luteolin on behavior, 5-MTHF levels, hippocampal neurogenesis, and folate brain transport of the CP. *In vitro* primary hippocampal neural stem cells (NSCs) cultured in media containing 10% CSF from each group of rats and choroid plexus epithelial cells (CPECs) cultured in media containing 20 μM luteolin were treated with 100 μM corticosterone and 40 mg/ml D-galactose. We found that aged rats exposed to CUMS showed a significantly reduced sucrose preference, decreased locomotion activity in the open field test and accuracy of the Morris water maze test, increased immobility time in the forced swimming test, accelerated dysfunctional neurogenesis and neuronal loss in the dentate gyrus of LOD rats, as well as decreased CSF and hippocampus 5-MTHF levels, and zona occludens protein 1 (ZO-1), proton-coupled folate transporter (PCFT), and reduced folate carrier (RFC) protein levels. *In vitro* assays showed media containing 10% aged CSF or LOD+ Luteolin-CSF significantly increased the viability of CORT + D-gal-injured NSCs and alleviated dysfunctional neurogenesis and neuronal loss compared with the CORT + D-gal medium. However, media containing 10% LOD-CSF had no such effect. In the meantime, induction of CORT + D-gal significantly decreased the ZO-1, PCFT, RFC, and folate receptor alpha (FR-α) protein levels and transepithelial electrical resistance in rat CPECs. As expected, luteolin treatment was effective in improving these abnormal changes. These findings suggested that luteolin could ameliorate CUMS-induced LOD-like behaviors by enhancing the folate brain transport.

## Graphical Abstract

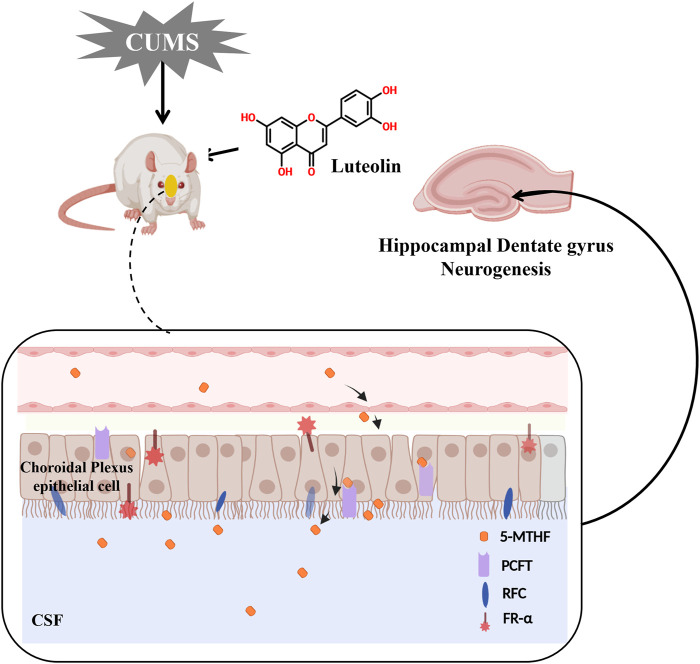

## Introduction

Age-related disorders currently represent one of the most important and challenging health problems worldwide. Late-onset depression (LOD) is defined as depression manifesting for the first time in life after the age of 65. Its clinical manifestations are a persistently depressed mood or anhedonia (i.e., loss of pleasure in normal daily activities), with varying degrees of somatic symptoms, cognitive impairment, delusions, and psychomotor retardation (Tam and Lam, 2013). The prevalence of clinically significant depressive syndromes in LOD ranges from 9% to 18% and shows incidence rates of 19% (Naismith et al., 2012). LOD not only significantly affects the quality of life of elderly individuals but also entails heavy financial and psychological burdens for their families and society.

Hippocampal damage is a well-established mechanism of depression (Kopp-Bigault and Walter, 2019), and hippocampal atrophy is also suggested to be a clinical hallmark of LOD (Roose and Schatzberg, 2005). Hippocampal neurogenesis and neuronal apoptosis are both important causes of hippocampal atrophy. In addition, the cerebrospinal fluid (CSF) is intrinsically related to hippocampal neurogenesis. Substances in the CSF can act directly on the hippocampus and affect its structure and function, including the regulation of hippocampal neurogenesis (Holzel et al., 2018).

Folate deficiency has attracted the attention of a growing number of scholars in the field of LOD pathogenesis. Studies have found that low folate levels in people over 65 years of age are associated with an increased risk of depression over the following 2–3 years (Kim et al., 2008), and folate deficiency might increase the risk of LOD in older adults (Araujo et al., 2015). Sufficient folate is particularly essential for neurodevelopment (Wang et al., 2019), and folate is a critical modulator of hippocampal neurogenesis (Naewla et al., 2019). *In vitro* studies have shown that environmental intervention, such as folate deficiency, and exposure to the dihydrofolate reductase inhibitor methotrexate decreases the proliferation of hippocampal neural stem cells and stagnates them in the S-phase, accompanied by an increased rate of apoptosis of newborn neurons (Kruman et al., 2005; Naewla et al., 2019). Previous studies have also found that in the presence of folate deficiency, the number of proliferating cells in the hippocampal dentate gyrus is significantly diminished, and hippocampal neural plasticity is altered, leading to hippocampal volume atrophy, which affects memory and cognitive function (Pan et al., 2017). Indeed, a reduction in the hippocampal volume is a typical clinical manifestation of LOD.

Folate is mostly metabolized by nervous tissue, and 5-methyltetrahydrofolate (5-MTHF) is the principal circulatory form of folate in the blood and CSF, which is transported across biological membranes into the central nervous system *via* the choroid plexus (CP). Neurons acquire 5-MTHF mainly through the blood–CSF barrier, whereas the uptake of 5-MTHF in the brain microvasculature through the blood–brain barrier is almost negligible (Spector and Johanson, 2006). Folate transport across biological membranes is mediated by three main pathways: the folate receptor alpha (FR-α), the proton-coupled folate transporter (PCFT), and the reduced folate carrier (RFC) pathways (Alam et al., 2020a). FRα and PCFT are ubiquitously expressed in the CP, have a very high affinity for 5-MTHF, and can transport 5-MTHF from the blood into epithelial cells at low folate levels. RFC is located at the apical part of the CP epithelium (facing the CSF) and comprises a low-affinity transport system that works only at relatively high folate concentrations (Ramaekers and Blau, 2004), which exports 5-MTHF aggregated in the CP epithelium to the CSF. Notably, the RFC is also located on the axons and dendrites of neurons and is the main mechanism of 5-MTHF ingestion by neurons. Consequently, the entry of folic acid into the central nervous system through the blood–CSF barrier is based mainly on the CP and the folate receptors and transporters on neurons (Djukic, 2007). Disruption of CP polarity disruption and transport dysfunction will alter the composition of the CSF (also including changes in the content of 5-MTHF in the cerebrospinal fluid), which in turn will affect hippocampal neurogenesis (Spector and Johanson, 2006; Alam et al., 2020a).

Currently, folate booster therapy is widely used in clinical practice to treat LOD, and folate is even added in food to increase folic acid levels (Payne et al., 2009; Freeman et al., 2010). Nevertheless, the process of absorption and biotransformation of folic acid to its active form (5-MTHF) is saturated at doses in regions of 200–400 μg of folic acid (Patel and Sobczynska-Malefora, 2017). The limitation of the metabolic process results in an inability to metabolize high doses of folic acid, leading to the accumulation of unmetabolized folic acid in the circulation. Elevated and prolonged exposure to unmetabolized folic acid might cause toxic effects in older women (Wansu et al., 2019). Excessive folic acid intake might result in, or exacerbate, the neurological damage associated with vitamin B12 deficiency (Powers, 2007). In terms of folate metabolism in neural tissues, excessive folate supplementation contributes to significant inhibition of the folate transporter expression; folate receptors have a much higher affinity for synthetic folate than 5-MTHF, and excessive supplementation of synthetic folate instead inhibits the 5-MTHF transport to brain tissues (Ashokkumar et al., 2007). In addition, CSF levels might be inadequate even when plasma folate levels are normal (Serot et al., 2001). Consequently, it is worthwhile investigating how to improve the 5-MTHF brain transport, enhance folate utilization in neuronal cells, and promote hippocampal neurogenesis.

**FIGURE 1:**
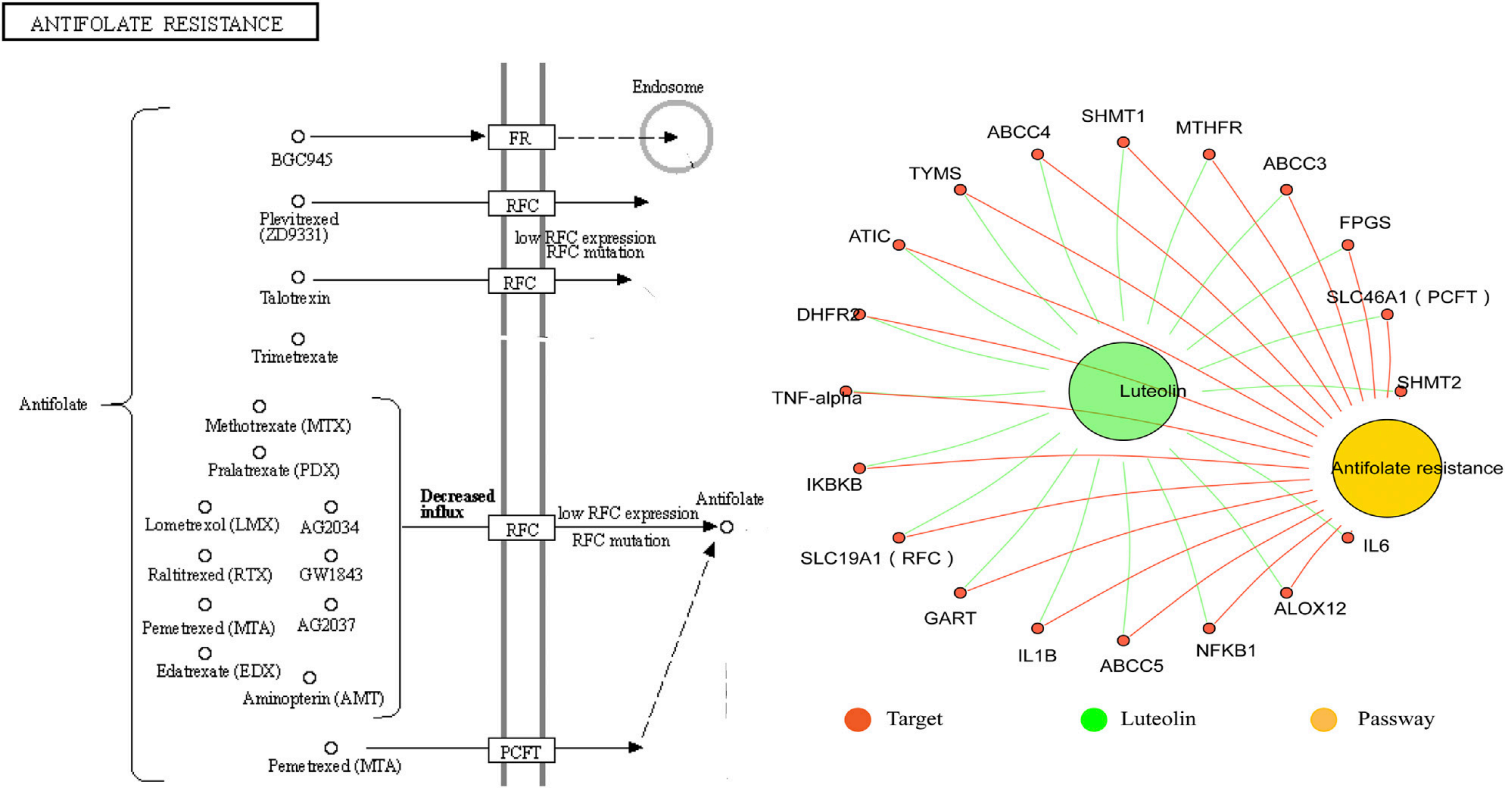
Association of the anti-folate resistance pathway with luteolin. The antifolate resistance pathway and target proteins of luteolin were downloaded from the herb database (http://herb.ac.cn/). The “Luteolin-target-pathway” network was structured by the Hiplot website (https://hiplot.com.cn/).

Luteolin (3′,4′,5,7-tetrahydroxyflavone) is a natural flavone compound that exists as a glycosylated form in many plants, including fruit, vegetables, and herbs. Conformational studies have shown that the existence of the hydroxyl portion of the luteolin structure at the carbon 5, 7, 3′, and 4′ positions and the 2–3 double bonds are associated with its multiple pharmacological effects (Lin et al., 2008; López-Lázaro, 2009). In early research, luteolin was observed to play an integral function as an anti-inflammatory agent in central nervous system diseases; however, more recent research showed that luteolin can improve central nervous system diseases through different mechanisms (Ashaari et al., 2018). For example, luteolin has good antidepressant effects and enhances the proliferation of hippocampal dentate gyrus neurons (Ishisaka et al., 2011; Zhou et al., 2019). In addition, we mined the potential targets of luteolin in the herb database (http://herb.ac.cn/) and found that luteolin is closely associated with anxiety disorders, depressive disorders, cognition disorders, and presenile dementia. Kyoto Encyclopedia of Genes and Genomes (KEGG) enrichment analysis of luteolin’s target proteins showed that it is involved in the anti-folate resistance pathway, in which PCFT and RFC are its potential targets, as shown in Figure 1.

The present study is based on the key role of luteolin in promoting hippocampal neurogenesis of central nervous system disorders and targeting folate transporters. Questions are raised as to whether luteolin might improve LOD by enhancing the brain transport of folate, increasing 5-MTHF levels in the CSF and hippocampus, and promoting hippocampal neurogenesis. To address this challenge, 20–21-month-old male Wistar rats were exposed to the chronic unpredictable mild stress (CUMS) protocol for 6 consecutive weeks to establish an LOD model. Rats in each group were given the corresponding drugs *via* intragastric administration. After 6 weeks, the animals were subjected to a series of behavioral tests. Plasma, CSF, and hippocampal concentrations of 5-MTHF were determined. The number of hippocampal 5-bromo-2-deoxyuridine (BrdU)/doublecortin (DCX), Ki-67/Nestin, and neuronal nuclear antigen (NeuN)-positive cells and the levels of zona occludens protein 1 (ZO-1), PCFT, RFC, and FR-α proteins in the CP were determined. *In vitro* high-concentration corticosterone (CORT, 120 μM) combined with D-galactosamine (D-gal, 40 mg/ml) was used to simulate LOD. We then explored the effects of LOD+ Luteolin-CSF on the proliferation and differentiation of primary hippocampal neural stem cells (NSCs) under CORT + D-gal conditions, observed the effects of luteolin on the levels of ZO-1, PCFT, RFC, and FR-α proteins, and determined the change of transepithelial electrical resistance (TEER) in primary choroid plexus epithelial cells (CPECs) under CORT + D-gal conditions.

## Materials and Methods

### Animals

For the analysis, 8–9-month-old male Wistar rats were purchased from Beijing Vital River Laboratory Animal Technology Co., Ltd. {Beijing, China [License No. SCXK (Jing) 2016-0011]} and raised for 12 months under standard conditions with free access to food and water. All efforts were made to minimize animal suffering, and all experimental procedures and protocols were approved by the Experimental Animal Ethics Committee of the Guangzhou University of Chinese Medicine in China.

### Generation of CUMS Models of Depression

The CUMS model of depression has been widely used as an experimental tool to investigate human psychopathology. Its reliability, practicability, and validity have been repeatedly demonstrated previously (Willner, 1997). The CUMS protocol was based on our previous studies (Zeng et al., 2022). For the protocol, 20–21-month-old rats were caged individually and underwent the sucrose preference test (SPT). The rats with the following performance(s) were omitted: low sucrose preference (less than 60%), positional preference (preferred to drink liquid from a fixed location), drinking too little (drinking neither sucrose solution nor pure water), or excessive drinking (total liquid consumption more than twice the average total liquid consumption of all rats).

A total of 60 eligible rats were randomly divided into the following three groups by body weight and SPT. 1) Rats housed three per cage and underwent normal feeding without any stressors as an AGED group [received normal saline (4 ml/kg) intragastrically]. 2) Rats housed individually and exposure to CUMS as a LOD group [received normal saline (4 ml/kg) intragastrically]. 3). Rats administered intragastrically with luteolin based on CUMS as a LOD + Luteolin group [Luteolin (purity ≥98% determined by high-performance liquid chromatography) was purchased from Nanjing Dilger Medical Technology Co. Ltd. (Nanjing, China) 25 mg/kg]. The stress protocol was as follows: electric shocks (1 mA, 2 s/time, 10 time/5min); 5 min of thermal swimming at 45°C; white noise (85 dB, 5 h); 5 h of stroboscopic illumination (300 flashes/min); 10 h of exposure to a soiled cage; 10 h of being paired with three other stressed animals; 12 h of restraint; water deprivation for 24 h, and food deprivation for 12 or 24 h. In brief, rats were randomly exposed to 1–2 stressful stimuli once a day for 6 weeks, without stressors repeated for more than 3 consecutive days.

### Behavior Test

#### SPT

The SPT was performed at the end of the 6-week CUMS exposure. The SPT involved four phases: sucrose training (48 h), baseline testing (36 h), food and water deprivation (24 h), and sucrose preference testing (12 h). During the sucrose training, baseline testing, and sucrose preference testing phase, rats were given the same amount of pure water and 1% sucrose solution. All rats were solitary-housed during the SPT. The volume of each solution that was consumed was then recorded to calculate the sucrose preference using the following formula: sucrose preference (%) = sucrose solution consumption/total liquid intake × 100.

#### Open Field Test

To assess the locomotion activity and exploration in a novel environment, the OFT was performed. Before the test, the rats habituated 1 h in the sound-free and dark behavioral test chamber. At the beginning of the test, rats were placed individually in the center of an open black box (100 cm × 100 cm × 48 cm) and allowed to explore freely for 5 min. The total distances by rats were recorded by using an automatic analysis system (Flydy Co., Ltd., Guangzhou, China). The apparatus was cleaned with detergent before each test session to remove any olfactory cues.

#### Forced Swimming Test

Before testing, experimental rats were acclimated in the behavioral test chamber for 1 h. Each rat was placed in a cylinder (30 cm diameter, 100 cm height) containing 35 cm of water (25 ± 1°C) and forced to swim for 6 min. The duration of the immobile posture (rats floating on the water surface with limbs immobilized or forepaws and tail gently padded to keep head above the water) was considered as the immobility time. The immobility time among the last 4 min was recorded to assess the desperation behavior in rats. The data were analyzed by researchers blinded to the experimental design.

#### Morris Water Maze Test

For analyzing the ability of rats to acquire learning and memory, we performed the MWM test (Hu et al., 2017). The MWM apparatus consisted of a circular basin (diameter 130 cm, height 55 cm, and divided into four quadrants: NW, NE, SE, and SW.) which contained about 45 cm of water (25 ± 1°C). This test included two periods: the place navigation test and the spatial probe test. The escape platform (learning place, invisible condition, and diameter 10 cm) was placed in the SW quadrant of the circular basin and 1 cm below the water surface during the 5-day place navigation test. The rats were placed into the water in the order of SW, NW, NE, and SE and were allowed to stay for 10 s after climbing onto the hidden platform. If failed to find the platform within 120 s, the rats were guided to the platform and were allowed to stay for 10 s. Rats were trained four times a day with an interval of 15 min each and were paid attention to the change order of entering daily. In the spatial probe test, the invisible platform was removed, and then, the rats were placed into the water facing the wall of the pool at the NE quadrant, and the percentage of their swimming distance and time in the platform quadrant was measured for 120 s.

#### CSF Sample Preparation

CSF samples were collected 24 h after the MWM test. After the rats were anesthetized (30 mg/kg, pentobarbital, intraperitoneal injection), their heads were fixed. The cerebellomedullary cistern was punctured with an intravenous infusion needle (0.45^#^) attached to a 1-ml syringe to collect the CSF, followed by centrifugation at 2,000 × g for 15 min at 4°C. The supernatants (containing the CSF) of six rats in each group were collected for 5-MTHF testing; the remaining CSF was collected for cell culture. After CSF collection, the rats were sacrificed for brain tissue sampling.

#### Enzyme-Linked Immunosorbent Assay

The hippocampus and CP sections of rats were sonicated in radioimmunoprecipitation assay (RIPA) buffer containing protease inhibitors. The plasma and CSF were taken out from −80°C. 5-MTHF was quantified using enzyme-linked immunosorbent assay kits (Jiangsu Meimian Industrial Co., Ltd., China), according to the manufacturer’s protocol. Detection limits were 48 ng/ml. A microplate reader was used for detection (3903-2010, Bio-Rad Company, USA).

### Tissue Preparation and Immunofluorescence

#### BrdU and DCX Double Labeling

Based on earlier studies (Huang et al., 2020), four rats in each group were injected intraperitoneally with 5-bromo-2-deoxyuridine [BrdU, Sigma (three injections, 200 mg/kg, 4 h apart)] 1 week before the sampling date. One week later, the brain tissues were dissected out and fixed for 24 h in 4% paraformaldehyde at 4°C and then immersed in 30% sucrose until sinking at the bottom of the tube. Serial sections (40 μm) containing the dentate gyrus (DG) of the hippocampus (2.7–6.7 mm from the coronal sulcus) were cut and stored at −80°C. BrdU/doublecortin (DCX) double labeling was performed to label the newly formed precursor neurons proliferating and differentiating from NSCs in the DG. Sections were treated with 2 M HCl for 20 min at 37°C, then neutralized with borate buffer (0.1 M, pH 8.4) for 15 min, blocked in 5% goat serum [(Beyotime, Beijing, China) containing 0.3% Triton X-100] at room temperature for 2 h, and incubated with rat anti-BrdU (1:200, ab6326, Abcam, Cambridge, UK) and rabbit anti-DCX (1:200, ab18723, Abcam) at 4°C overnight. After washing in Tris-buffered saline (TBS) with 0.01% Tween-20, the sections were incubated with AlexaFluor^®^ 594 goat anti-rat IgG (1:500, ab150160, Abcam) and AlexaFluor^®^ 488 goat anti-rabbit IgG (1:500, ab150077, Abcam) at 37°C for 2 h. After washing and 4′,6-diamidino-2-phenylindole (DAPI) staining, images were captured by using a laser confocal microscope (LSM800, ZEISS, Oberkochen, Germany). The number of BrdU/DCX double-positive cells and the number of nuclei were calculated by Photoshop (Adobe Photoshop Inc., San Jose, CA, United States). The proportion of BrdU/DCX double-positive cells (%) was calculated as follows: the number of BrdU/DCX double-positive cells/total number of nuclei × 100.

#### Ki67 and Nestin Double Labeling

In the Ki67 and Nestin staining stage, sections were incubated with a rabbit anti-Ki67 antibody (1:200, 9129, CST) and mouse anti-Nestin (1:200, ab6142, Abcam) at 4°C overnight. After washing in TBS with 0.01% Tween-20, the sections were incubated with AlexaFluor^®^ 488 goat anti-mouse IgG (1:500, ab150133, Abcam) and AlexaFluor^®^ 594 goat anti-rabbit IgG (1:500, ab150080, Abcam) at 37°C for 2 h. The other steps and parameters were consistent with the aforementioned staining except for HCL treatment and borate buffer neutralization similar to BrdU/DCX labeling. The number of Ki67/Nestin double-positive cells was recorded.

#### NeuN Single Labeling

NeuN was used to label and count mature neurons in the DG, following the same procedure as Ki67/Nestin double labeling. Sections were incubated with a rabbit anti-NeuN antibody (1:1,000, ab177487, Abcam) overnight at 4°C and AlexaFluor^®^ 488 goat anti-rabbit IgG (1:500, ab150077, Abcam) at 37°C for 2 h. The proportion of NeuN-positive cells (%) was calculated as follows: NeuN-positive cell number/total number of nuclei (DAPI) × 100.

#### ZO-1, FR-α, RFC, and PCFT Single Labeling

Other four rats in each group were perfused transcardially with 4% paraformaldehyde. The brain tissues were dissected out, and fixation was continued for 24 h in 4% paraformaldehyde at 4°C and then immersed in 30% sucrose until sinking at the bottom of the tube. Serial sections of 10 μm thick slices were cut through the entire choroid plexus (-4.5–1.2 mm from the fontanel) and stored at −80°C. The sections were incubated respectively with rabbit anti-ZO-1 (1:50, 61-7300, Invitrogen), rabbit anti- FR-α (1:200, PA5-42004, Invitrogen), rabbit anti- RFC (1:200, ab62302, Abcam), and rabbit anti- PCFT (1:200, ab25134, Abcam). The other steps and parameters were consistent with those previously mentioned. The mean fluorescence intensities were calculated by ImageJ software.

#### Western Blotting

The protein blots were prepared according to the conventional procedures of Western blot analysis. Hippocampal and CP tissues were collected from other four rats per group, followed by washing, homogenization, and lysing in RIPA buffer containing protease inhibitors. After conventional protein extraction from the hippocampal and CP tissues, the total protein concentration was measured, followed by adding 30 *μ*g total protein to 1/4 volume of 5 × SDS-PAGE (P0015, Beyotime Biotechnology) sample buffer, boiling the protein sample for 10 min, and separating the proteins by SDS-PAGE. After transferring the separated proteins onto the PVDF membrane (IPVH00010, Millipore), the membrane was incubated with primary antibodies such as doublecortin (1:2,000), nestin (1:2,000), NeuN (1:2,000), ZO-1 (1:2,000), FR-α(1:2,000), RFC(1:2,000), PCFT (1:2,000), GAPDH (1:5,000), and β-actin (1:5,000) overnight at 4°C, and secondary antibodies (1:5,000; Abcam) were incubated for 2 h at 37°C. The absorbance values of protein bands were scanned by using an Odyssey infrared gel imaging system (Affinity, USA). Densitometry was performed to quantify the signal intensity by ImageJ software (version 1.45 J; National Institutes of Health, Bethesda, MD, United States).

#### Isolation of Primary Hippocampal NSCs From the Hippocampus

Embryos were removed from etherized Wistar rats {obtained from the Laboratory Animal Center of Southern Medical University, Guangzhou, China [License No. SCXK (Yue) 2016-0041]} on embryonic days 16–18 under sterile conditions. Primary hippocampal NSC culturing conditions were performed, as described by Zeng et al., (2022).

#### Cell Counting Kit-8 Test

.Passage 1 NSCs were collected and divided into eight groups: the control group [cultured with the NSC medium (Dulbecco’s modified Eagle media/nutrient mixture F-12 containing 20 ng/L epidermal growth factor, 20 ng/L basic fibroblast growth factor, 2% B27, and 1% penicillin/streptomycin)], high-concentration corticosterone (CORT) and D-galactose (D-gal) (CORT + D-gal group) [cultured with the NSC medium containing 120 μM CORT (235135, Millipore) and 40 mg/ml D-gal (V900922, Sigma)], the AGED-CSF group [cultured with the NSC medium containing 10% CSF harvested from AGED rats (AGED-CSF)], the LOD-CSF group [cultured with the NSC medium containing 10% CSF harvested from LOD rats (LOD-CSF)], and the LOD+ Luteolin-CSF group [cultured with the NSC medium containing 10% CSF harvested from LOD+ Luteolin rats (LOD+ Luteolin -CSF)], the D-gal + AGED-CSF group [cultured with the NSC medium containing 10% CSF harvested from AGED rats (AGED-CSF) and 40 mg/ml D-gal], the CORT + D-gal group + LOD-CSF group [cultured with the NSC medium containing 10% CSF harvested from LOD rats (LOD-CSF), 120 μM CORT and 40 mg/ml D-gal], and the CORT + D-gal group + LOD+ Luteolin-CSF group [cultured with the NSC medium containing 10% CSF harvested from LOD + Luteolin rats (LOD+ Luteolin -CSF), 120 μM CORT and 40 mg/ml D-gal]. Cell viability was detected using a cell counting kit-8 (CCK8). The specific experimental approach is as we have described previously (Zeng et al., 2022). The optical density (OD) was measured at 450 nm with a microplate reader (Bio-Rad, Hercules, CA, United States). The cell viability was calculated as follows: cell viability (%) = [OD (experimental)—OD (blank)]/[OD (CON)—OD (blank)] × 100%.

#### Immunofluorescence Analysis of Hippocampal NSCs

As we previously described (Zeng et al., 2022), NSCs were dispersed into single cells using Accutase TM, resuspended in the differentiation culture medium (Neural Basal containing 10% FBS), and seeded into 15-mm confocal dishes (5000 cells/dish). After 48 h, CORT, D-gal, and CSF were added separately according to the grouping. The detailed grouping was the same as CCK-8. The cells were continued to be cultured for 48 h, and then, 10 μM BrdU was added to each dish. After 48 h, the cells were fixed with 4% paraformaldehyde and labeled with BrdU/DCX. The number of BrdU/DCX double-positive cells and the number of nuclei were counted as mentioned earlier.

#### Primary Culture of CPECs

For the procedure, 8–9-month-old male Wistar rats were purchased from the Laboratory Animal Center of Southern Medical University, Guangzhou, China). The CP was separated from the brains and homogenized into CP epithelial cell (CPEC) suspensions, as described (Monnot and Zheng, 2013; Lallai et al., 2020). Simply, CP tissues were minced into small pieces (less than 1 mm cube) and added in 1 mg/ml Type I collagenase (17100-017, Gibco). The solution was then pipetted gently up and down with a Pasteur pipette to dissociate the tissues and incubated at 37°C for 10 min. Then, CPEC medium [DMEM+10% FBS+1% penicillin–streptomycin + rat epidermal growth factor (20 ng/ml)] terminated the digestion. The solution was filtered through a 200-mesh cell sieve to obtain a single-cell suspension and centrifuged at 260 × g for 5 min. The supernatant was discarded, and the CPECs were resuspended with the CPEC medium in a humidified incubator with 95% air/5% CO_2_ at 37°C incubation. After 10 h, nonadherent cells were collected and seeded into 35-mm culture dishes at a density of 3 × 10^5^ cells/mL and incubated in an incubator. The CPEC medium was replaced with fresh medium every 3 days.

#### Immunofluorescence Analysis of CPECs

Passage 1 CPECs were collected and seeded into a confocal culture dish at a density of 2 × 10^5^ cells/mL. When cells reached ∼90% confluency as a monolayer, CORT, D-gal, and luteolin were added separately, according to the grouping. The detailed grouping is as follows: the control group (cultured with the CPEC medium), D-gal group (cultured with the CPEC medium containing 40 mg/ml D-gal), CORT + D-gal group (cultured with the CPEC medium containing 120 μM CORT and 40 mg/ml D-gal), and the CORT + D-gal + Luteolin group (cultured with the CPEC medium containing 120 μM CORT, 40 mg/ml D-gal, and 20 μM luteolin). The cells were continued to be cultured for 72 h and then fixed with 4% paraformaldehyde and labeled with ZO-1, FR-α, RFC, and PCFT. The steps used were the same as those mentioned earlier.

#### Transepithelial Electrical Resistance Measurement

TEER was measured in Ohms using a Millicell-ERS resistance apparatus (ERS2, Millipore, USA). Passage 1 CPECs were collected and seeded into a collagen-coated precoated transwell^®^ at a density of 2 × 10^5^ cells/mL and continued to be cultured for 48 h. After 48 h, the medium was replaced according to each group, respectively, for another 72 h. Wells without cells were used as blank standard. The detailed grouping was the same as immunofluorescence analysis of CPECs. After culture for 72 h, TEER was measured every day with a Millicell-ERS resistance apparatus (ERS2, Millipore, USA), and the changes of TEER under each intervention condition were dynamically observed.

#### Statistical Analyses

SPSS 25.0 software (IBM, Armonk, IL, United States) was used to perform all statistical analyses. Data normality was determined using the Shapiro–Wilk normality test. Nonparametric tests are used if the normality test is not consistent with a normal distribution (SPT results). Data consistent with a normal distribution were analyzed with one-way ANOVA. The number of target platform crossings in MWT exhibited heterogeneity of variance, while the other results exhibited homogeneity of variance. For pairwise comparisons, the Games-Howell method was used to assess results with heterogenous variance (the number of target platform crossings in MWT), and the least significant difference test was used to assess results with homogenous variance (other results). The Morris water maze escape latency was analyzed by repeated measures analysis of variance. Data are expressed as the mean ± standard error of the mean (SEM). The significance level was set at *p* < 0.05. GraphPad Prism 8.0.2 (GraphPad Software Inc., CA, United States) was used to dew plots in the study.

## Results

### Effects of Luteolin on Behavioral Changes

**FIGURE 2:**
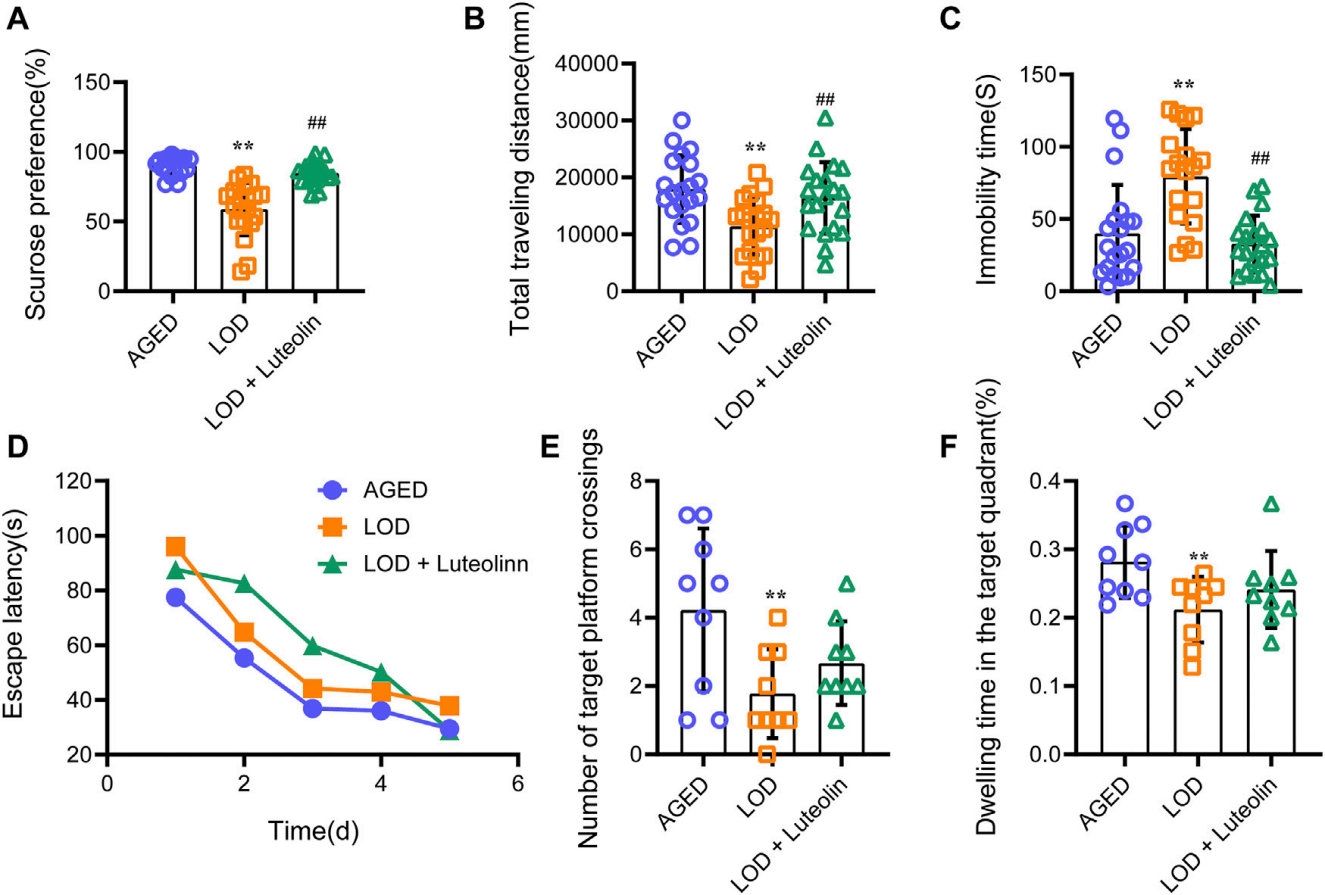
Effects of luteolin on the behavior of LOD rats. **(A)** Results of the SPT (*n* = 18–20); **(B)** total distance in the open field test (*n* = 18–20); **(C)** immobility time in the FST (*n* = 18–20); **(D)** Escape latency in the MWM test (*n* = 9); **(E)** Number of target platform crossing in the MWM test (*n* = 9); **(F)** Dwelling time in the target quadrant in the MWM test (*n* = 9). SPT results were not consistent with a normal distribution and used non-parametric tests for comparisons. Data consistent with a normal distribution were analyzed using one-way analysis of variance. For pairwise comparisons, the Games-Howell method was used to assess results with heterogenous variance (number of target platform crossings in MWT), and the least significant difference test was used to assess results with homogenous variance (other results). The MWM escape latency was analyzed by repeated-measures analysis of variance. Data are expressed as the mean ± SEM, *p < 0.05, **p < 0.01, vs. AGED; ^#^ *p* < 0.05, ^##^ *p* < 0.01, vs. LOD.

Following exposure to CUMS for 6 weeks, the behavioral tests were performed. As shown in Figures 2A–C, compared with the AGED group, the LOD group exhibited significantly reduced sucrose preference and the total traveling distance in OFT and an increased immobility time in the FST. The results of the LOD + Luteolin group were normalized to those of the AGED group and were significantly different from those of the LOD group. As shown in Figures 2D–F, in the MWM test, the LOD group took more time (increased latency time) to reach the hidden platform than that of the AGED group. The LOD + Luteolin group showed enhanced memory function, as indicated by the rats taking less time to arrive at the hidden platform, compared with that of the LOD group. Furthermore, a probe test of the MWM test demonstrated that luteolin increased the number of platform crossings and time spent in the target quadrant compared with those of the LOD group.

### The Effect of Luteolin on 5-MTHF in the Plasma, CSF, and the Hippocampus

**FIGURE 3:**
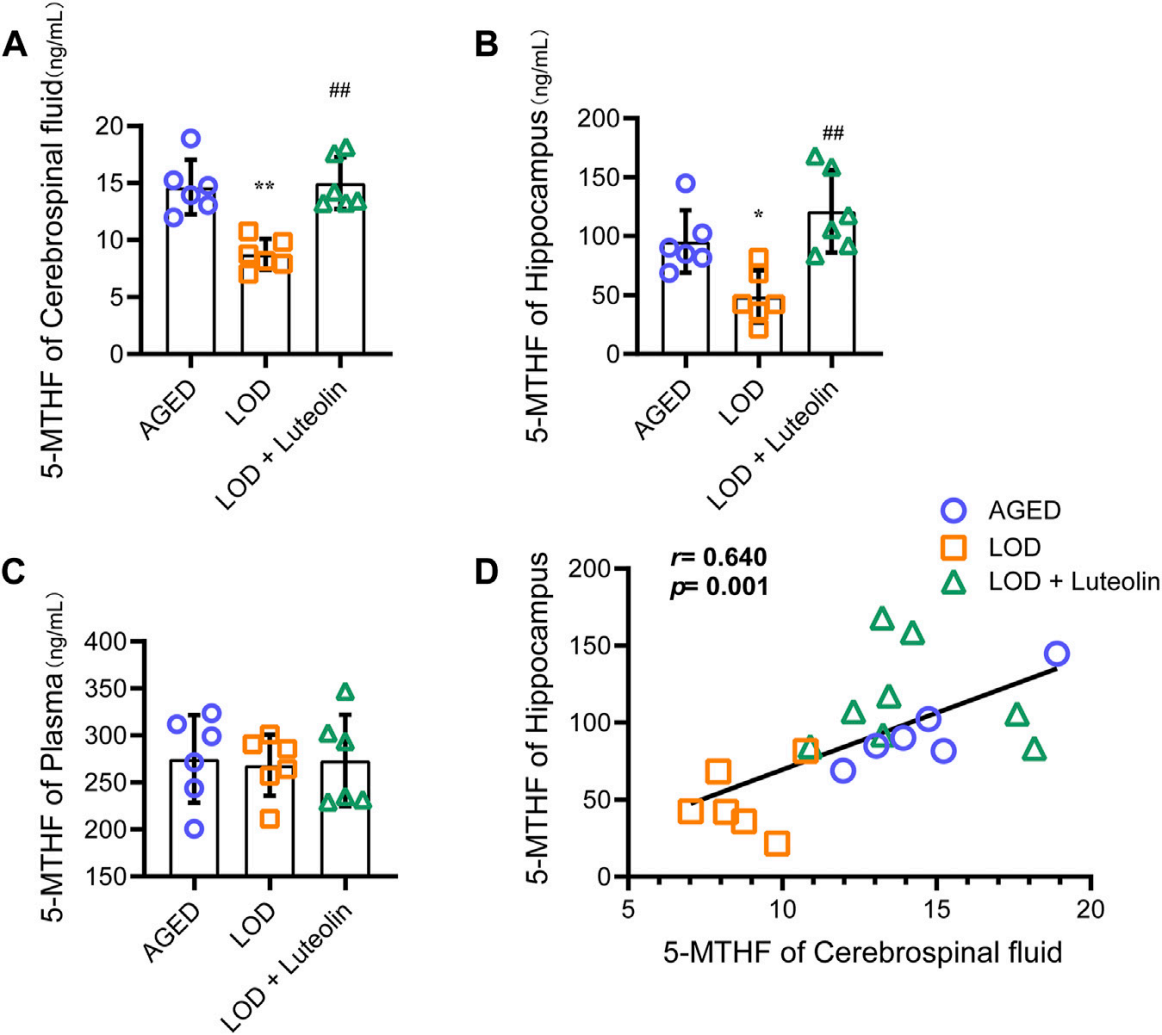
Effects of luteolin on the plasma, CSF, and hippocampus 5-MTHF levels in LOD rats. The levels of the plasma, CSF, and hippocampus 5-MTHF are shown **(A–C)** (*n* = 6). **(D)** Correlation analysis of the CSF and hippocampus 5-MTHF levels (*n* = 6). Data in each group were consistent with a normal distribution (Shapiro-Wilk test) and exhibited homogeneity of variance; one-way analysis of variance was then used for comparisons. Data are expressed as the mean ± SEM, *p < 0.05, **p < 0.01, vs. AGED; ^#^ *p* < 0.05, ^##^ *p* < 0.01, vs. LOD.

Next, we analyzed the plasma, CSF, and hippocampus 5-MTHF levels. As shown in Figures 3A,B, compared with those in the AGED group, a significant reduction in the CSF and hippocampus 5-MTHF levels was observed in the LOD group. CSF and hippocampus 5-MTHF levels were higher in the LOD + Luteolin group than in the LOD group. Curiously, no statistical differences in plasma 5-MTHF levels were noted among the groups (Figure 3C). Through correlation analysis, we found that the CSF 5-MTHF levels correlated positively within the hippocampus (Figure 3D).

### Effect of Luteolin on the Relative Number of Neurons in the Hippocampus DG Region in LOD Rats

**FIGURE 4:**
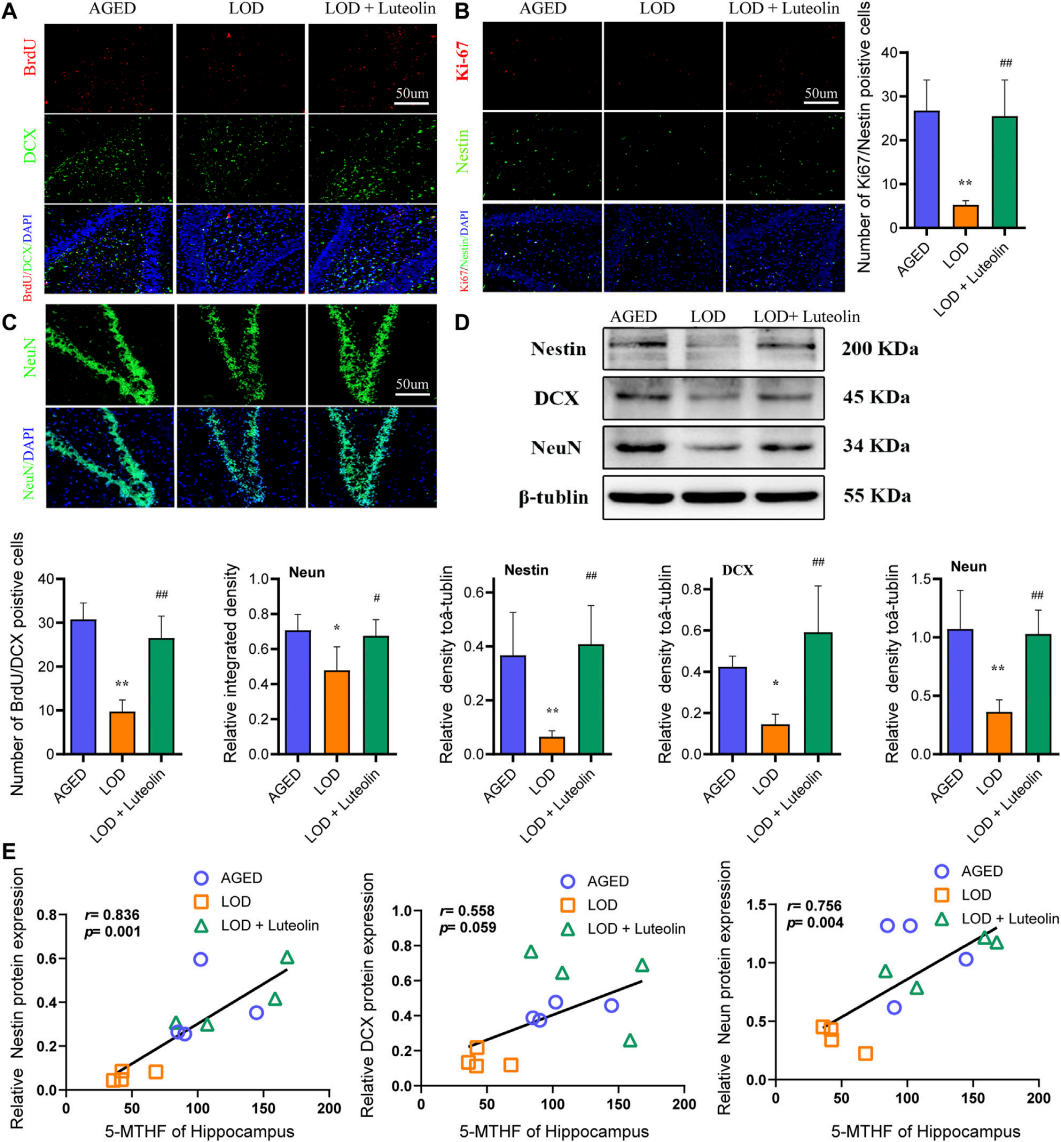
Effects of luteolin on the numbers of BrdU/DCX, Ki-67/Nestin, and NeuN-positive cells in the hippocampus. Quantitative analysis of DCX/BrdU double-positive cells, Ki-67/Nestin double-positive cells, and NeuN-positive cells in the hippocampus DG region **(A–C)** (*n* = 4), Magnification ×10. Representative Western blotting images of Nestin, DCX, and NeuN protein levels in the hippocampus **(D)** (*n* = 4). Correlations between 5-MTHF and NeuN levels in the hippocampus, between 5-MTHF and DCX levels in the hippocampus, and between 5-MTHF and Nestin protein levels in the hippocampus **(E)** (*n* = 4). Data in each group were consistent with a normal distribution (Shapiro–Wilk test) and exhibited homogeneity of variance; one-way analysis of variance was then used for comparisons. Data are expressed as the mean ± SEM, *p < 0.05, **p < 0.01, vs. AGED; ^#^ *p* < 0.05, ^##^ *p* < 0.01, vs. LOD.

Adult neurogenesis is a common feature of the dentate gyrus in mammals and is divided into three main parts: cell proliferation, neuronal differentiation, and cell survival. In the immunofluorescence experiment, BrdU/DCX and Ki67/Nestin double labeling new nerve cells and neurons were used to reflect the number of neural precursor cells in the DG region of the hippocampus. The decrease of positive cells indicated that the proliferation and differentiation ability of neural stem cells reduced neurogenesis dysfunction. As shown in Figures 4A,B, compared with the AGED group, the number of BrdU/DCX and Ki67/Nestin double-positive cells in the hippocampus DG region of the LOD group reduced significantly. In contrast to the results for the LOD group, luteolin increased the number of BrdU/DCX and Ki67/Nestin-positive cells considerably in the hippocampus DG region. This indicated that luteolin treatment could alleviate LOD-induced neurogenesis dysfunction in the hippocampus DG region. In addition, as shown in Figure 4C, we found that compared with those in the AGED group, the relative number of neurons in the hippocampus DG region of the LOD group was reduced significantly. This suggested that there is also a loss of mature neurons in the hippocampus DG region of the LOD group. Compared with those in the LOD group, luteolin dramatically increased the relative number of neurons, suggesting that luteolin treatment could resist neuronal reduction in the hippocampus DG region. Moreover, Western blotting analysis showed reduced levels of Nestin, DCX, and NeuN in the hippocampus DG region of the LOD group, which was enhanced in the hippocampus DG of the LOD + Luteolin group (Figure 4D). Through correlation analysis, we found that hippocampus 5-MTHF levels correlated positively with Nestin, DCX, and NeuN protein levels (Figure 4E).

### Effect of Luteolin-CSF on the Proliferation and Differentiation of CORT + D-Gal-Induced Primary Hippocampus NSCs

**FIGURE 5:**
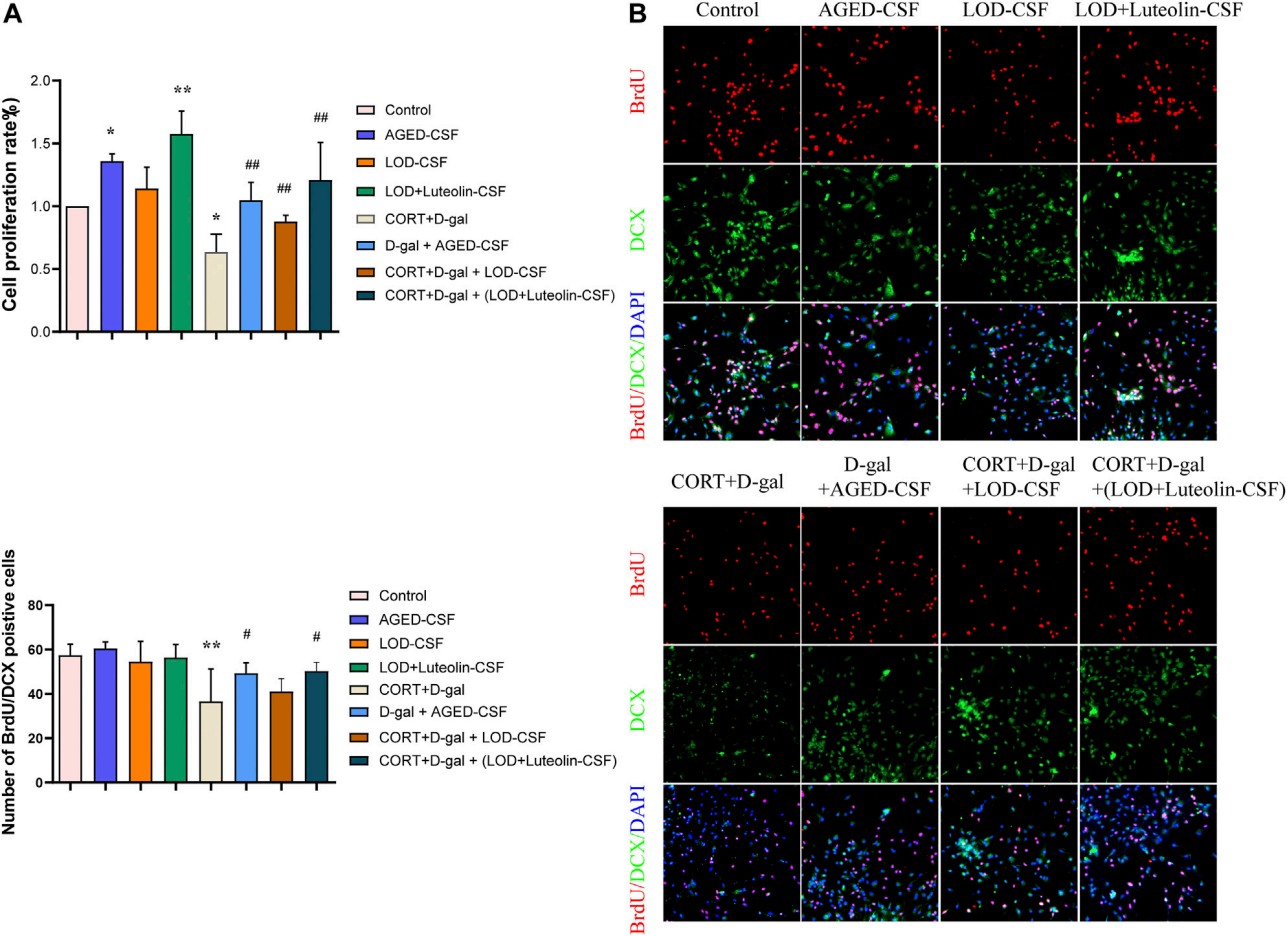
Effects of Luteolin CSF on the proliferation and differentiation of CORT + D-gal–induced primary hippocampus NSCs. **(A)** NSC viability was measured using a CCK-8 assay (*n* = 3). **(B)** DCX/BrdU double-positive NSCs were observed by immunofluorescence (*n* = 4). Data in each group were consistent with a normal distribution (Shapiro–Wilk test) and exhibited homogeneity of variance; one-way analysis of variance was then used for comparisons. Data are expressed as the mean ± SEM, *p < 0.05, **p < 0.01, vs. Control; ^#^ *p* < 0.05, ^##^ *p* < 0.01, vs. CORT + D-gal.

Chronic exposure of rodents to high concentrations of CORT is widely used to induce depression-like behavior and neurochemical changes associated with major depressive disorder symptoms. Similarly, CORT-induced cells have been extensively adopted as an *in vitro* model to investigate the impairment of neurons and depression-like syndromes. Chronic administration of D-gal induces brain aging and accelerates artificial senescence, which is used for a variety of anti-aging pharmacological research studies. In the present study, to investigate the effect on proliferation and differentiation of Luteolin-CSF in neurons, CORT + D-gal -injured NSCs were used. Cells were treated with different concentrations of CORT and D-gal and CSF from the different groups for 72 h, and the cell viability was determined using CCK-8 assays. According to the results (Figure 5A), treatment with 120 μM CORT and 40 mg/ml of D-gal for 72 h resulted in a decrease in the cell viability to approximately 40%; therefore, these concentrations were selected for subsequent experiments. In addition, we observed that 10% AGED-CSF and Luteolin -CSF significantly increased the viability of CORT + D-gal–injured cells compared with the CORT+ D-gal alone group. Moreover, 10% AGED-CSF and Luteolin -CSF promoted the viability of normally cultured NSC cells. Immunofluorescence analyses clearly showed that compared with those in the control group, the number of BrdU/DCX double-positive NSCs in the AGED-CSF and Luteolin-CSF groups increased significantly (Figure 5B). Compared with the CORT + D-gal -injured group, AGED-CSF and Luteolin -CSF treatment showed similar results. However, LOD-CSF did not increase the number of BrdU/DCX double-positive NSCs in the control or CORT + D-gal-injured groups. This indicated that AGED-CSF and Luteolin -CSF could alleviate CORT + D-gal–induced neurogenesis dysfunction in NSCs.

### Effects of Luteolin on the CP Structure and TEER of Rat CPECs

#### Effects of Luteolin on the CP Structure in the LOD Rats

**FIGURE 6:**
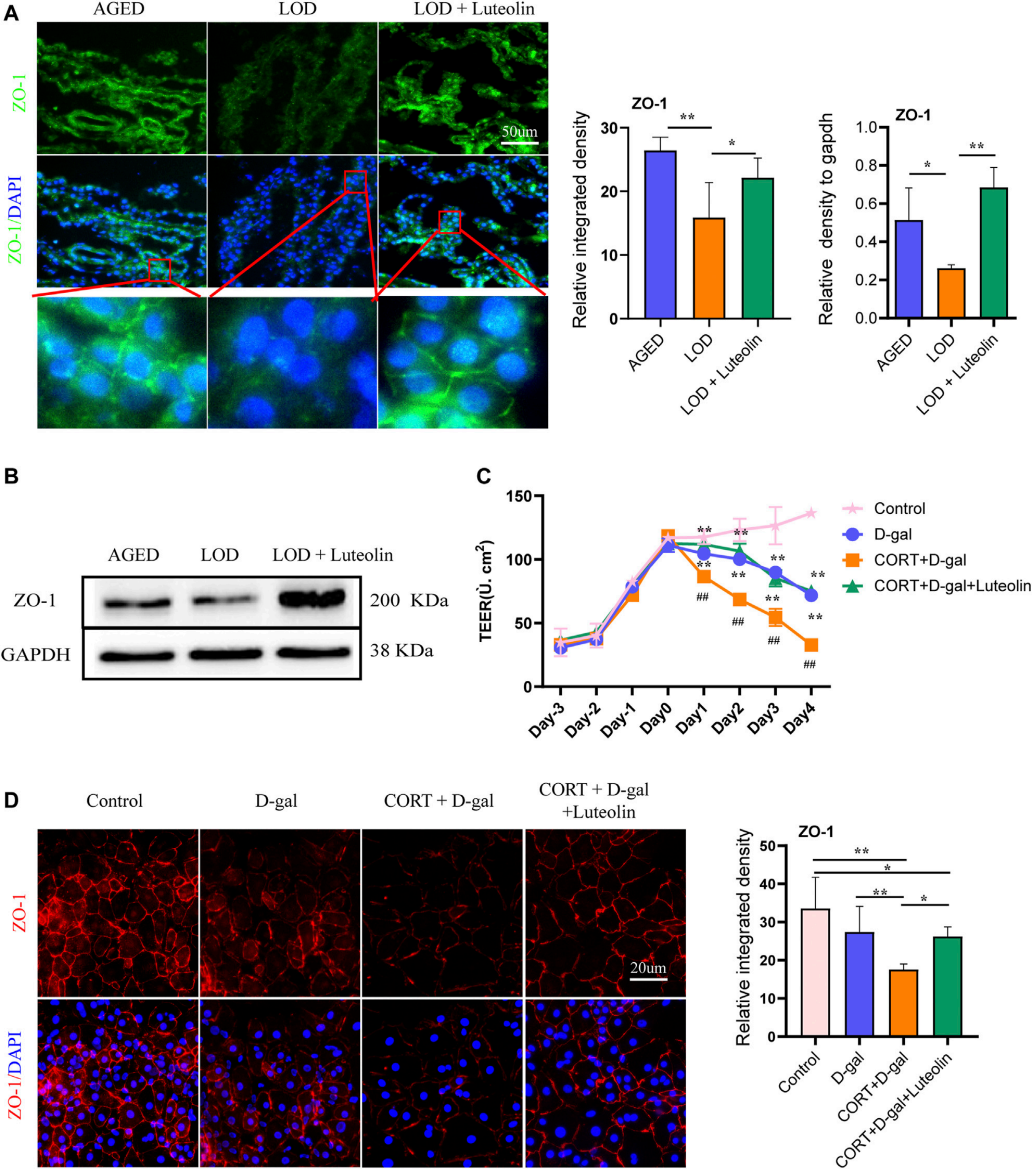
Effects of luteolin on rat CP polarity and rat CPEC polarity. Representative immunofluorescence image of ZO-1 levels in the rat CP **(A)** and CORT + D-gal-induced rat CPECs **(B)** (*n* = 4). TEER of CORT + D-gal-induced rat CPECs **(C)** (*n* = 3). Representative Western blotting images of ZO-1 protein levels in the CP (*n* = 4) **(D)**. Data in each group were consistent with a normal distribution (Shapiro–Wilk test) and exhibited homogeneity of variance; one-way analysis of variance was then used for comparisons. Data are expressed as the mean ± SEM, *p < 0.05, **p < 0.01 vs. LOD or CORT + D-gal. ^#^ *p* < 0.05, ^##^ *p* < 0.01, vs. Control.

Considering that the structural integrity of the CP plays an important role in folate trafficking, we next examined the tight junctions of rat CP tissues and CORT + D-gal-induced rats CPECs. The polarity of CORT + D-gal–induced rat CPECs was measured quantitatively using TEER. As shown in Figure 6A, compared with that in the AGED group, the structural integrity of the CP in the LOD group was destroyed, and the level of ZO-1 decreased. The LOD + Luteolin group showed a relatively complete CP structure compared with that in the LOD group and had an increased level of ZO-1 compared with that in the LOD group. This experiment was repeated, with similar results, using Western blotting (Figure 6B). Additionally, in the *in vitro* experiments, induction by CORT + D-gal significantly decreased the TEER and ZO-1 level in rat CPECs (Figures 6C,D). The opposite effects were observed under luteolin treatment.

#### Effects of Luteolin on the Levels of PCFT, RFC, and FR-α in the CP and CORT + D-Gal–Induced Rat CPECs

**FIGURE 7:**
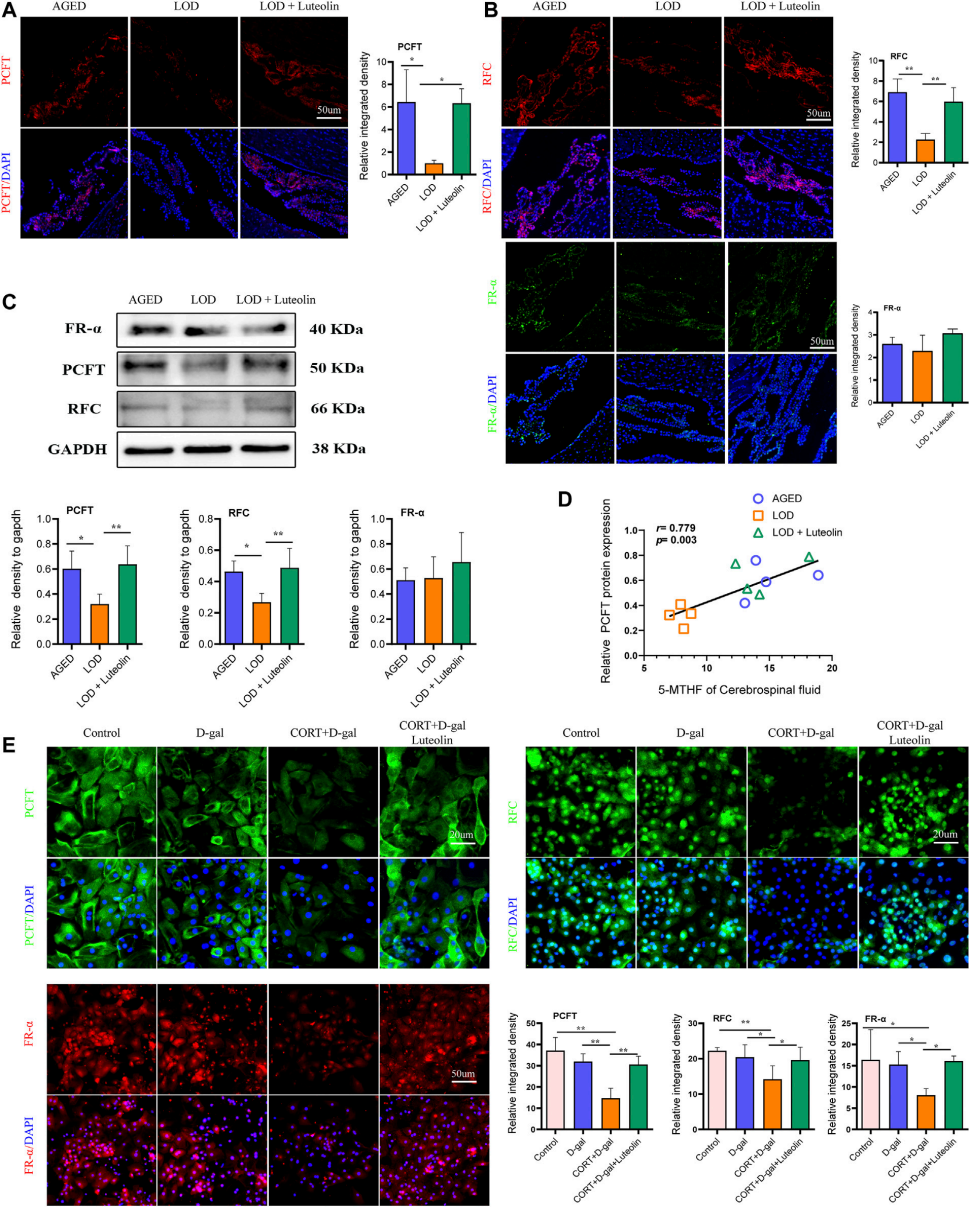
Effects of luteolin on the levels of PCFT, RFC, and FR-α in the CP and rat CPECs. Immunofluorescence images of PCFT, RFC, and FR-α in the CP **(A,B)** (*n* = 3). Representative Western blotting images of PCFT, RFC, and FR-α levels in the CP **(C)** (*n* = 4). Correlations between CSF 5-MTHF and PCFT protein levels **(D)** (*n* = 4), respectively. Immunofluorescence images of PCFT, RFC, and FR-α in rat CPECs **(E)** (*n* = 4). Data in each group were consistent with a normal distribution (Shapiro–Wilk test) and exhibited homogeneity of variance; one-way analysis of variance was then used for comparisons. Data are expressed as the mean ± SEM, *p < 0.05, **p < 0.01 vs. LOD or CORT + D-gal.

Based on immunofluorescence analysis (Figures 7A,B), the LOD group had lower PCFT and RFC protein levels in the CP than those in the AGED group. The LOD + Luteolin group showed elevated levels of PCFT and RFC compared with those in the LOD group. However, no statistical differences in the FRα protein expression were noted among the groups. Similarly, Western blotting further confirmed that luteolin treatment improved the levels of PCFT and RFC in the CP (Figure 7C). Correlation analysis showed that CSF 5-MTHF levels positively correlated with PCFT levels (Figure 7D). In addition, *in vitro* experiments demonstrated that induction by CORT + D-gal significantly decreased PCFT, RFC, and FRα protein levels (Figure 7E). The opposite effects were observed under luteolin treatment.

## Discussion

Depression manifesting for the first time in later life is termed LOD. In addition to persistent depression, fear, or anxiety, LOD is often associated with cognitive deterioration, and patients with LOD are very susceptible to the development of dementia (Notzon et al., 2016). In this study, natural aging superimposed on CUMS was utilized to simulate the symptoms and pathological modifications associated with LOD. We observed that LOD rats showed significant depression-like behavior accompanied by significant impairment of spatial memory, which were also consistent with the general clinical symptoms of LOD (Glover and Srinivasan, 2013).

The hippocampus is an essential neurogenic brain region, governing learning, situational memory, behavioral cognition, and emotion. Neurogenesis is crucial for memory and other cognitive functions, as well as the regulation of emotions. The hippocampal volume is diminished in patients with LOD, and the magnitude of volume variation was related to the duration and severity of depression (Lloyd et al., 2004; Banasr et al., 2021), and neuron apoptosis and neurogenesis disorders are both important causes of hippocampal volume reduction. In the present study, compared with the AGED group, we observed a substantial decrease in the level of NeuN protein and Ki-67/Nestin and BrdU/DCX double-positive cells in the hippocampal DG region of LOD rats. This reflects the loss of mature neurons in LOD rats, accompanied by a decrease in the number of neural stem cells and a diminished ability to differentiate. Conversely, administration of luteolin effectively maintained the expression of NeuN protein and increased the number of BrdU/Ki-67-labeled cells, which agrees with a previous study in which luteolin demonstrated a protective effect on hippocampal neurogenesis (Jang et al., 2010; Zhu et al., 2011).

Molecular signals in the CSF can directly target neural stem cells and have an effect on neurogenesis (Lugert et al., 2017); changes in the levels of several proteins in the CSF could predict different stages of neurogenesis in the DG of the adult hippocampus (Johansson et al., 2013). The CSF is mainly secreted by the CP, which is involved in stress-related diseases and is susceptible to the effects of aging (Serot et al., 2003; Sathyanesan et al., 2012). Once the CP is disturbed it might alter the CSF components. *In vitro* high CORT combined with D-gal was utilized to simulate LOD (Sterner and Kalynchuk, 2010; Ouyang et al., 2021). The results showed that CORT + D-gal significantly reduced the proliferation and differentiation ability of primary hippocampal NSCs, which were reversed by the rat luteolin CSF, suggesting that substances in the CSF could operate directly on the hippocampus and affect the hippocampal neurological function. Furthermore, we also found that even under D-gal conditions, the CSF in AGED rats promoted the proliferation and differentiation ability of primary hippocampal NSCs. This result also hinted that luteolin can play a role in promoting the proliferation of hippocampal neurons by altering the CSF components in LOD rats.

5-MTHF is the principal biologically active form of folate in the blood and CSF, which is metabolized predominantly by neural tissues and is transported across biological membranes into the central nervous system *via* the CP, with significant effects on mood and cognition. Folate deficiency is extremely common among people over 65 years of age. A population-based study indicated that folate deficiency was associated with an increased risk of LOD (Araujo et al., 2015). In this study, we found that the levels of 5-MTHF in the LOD CSF were significantly lower than those in the AGED group. The same results were observed in the hippocampus among the groups. Considering plasma 5-MTHF, Serot reported that patients with impaired cognition-related mental diseases had low levels of folate within their CSF. Furthermore, he emphasized that even when the blood folate level was normalized, CSF folate levels remained very low (Serot et al., 2001). Similar results were replicated in this study. Plasma folate levels were not significantly different among the groups. Therefore, the observed decrease of CSF-folate levels is not likely to be caused by a peripheral deficiency. It is suggested that normal absorption and metabolism of folate occurs in LOD rats but with a specific alteration of the CP transport function. Additionally, a positive correlation was observed between CSF 5-MTHF and hippocampus 5-MTHF levels, which tentatively suggested that CSF 5-MTHF might directly affect the hippocampal microenvironment and the 5-MTHF content in hippocampal neurons. As expected, luteolin significantly promoted the 5-MTHF content in the CSF and hippocampus of LOD rats, demonstrating that luteolin could effectively modify the obstructed folate brain transport.

Folic acid plays an essential role in the proliferation and differentiation of hippocampal NSCs. In the absence of folate, the number of proliferating cells in the hippocampal DG is significantly reduced, and hippocampal neurons undergo plasticity changes, leading to hippocampal volume atrophy, and memory and cognitive dysfunction (Kronenberg et al., 2008). There are numerous mechanisms by which the brain folate deficiency causes morphological changes in the hippocampus, such as the abnormal metabolism of homocysteine to glutamate (Hama et al., 2020), which in turn leads to enhanced excitotoxicity triggering neuronal apoptosis; improper folate intake and abnormal metabolism can reduce the genomic stability and cause neuronal apoptosis. However, folic acid deficiency is most likely to damage rapidly dividing cells. A study showed that folate deficiency causes HT-22 hippocampal neuronal cell cycle arrest in the G0/G1 phase and increases apoptosis (Yang et al., 2016). The results of the correlation analysis in this study showed that the 5-MTHF content in the hippocampus correlated positively with the levels of Nestin, DCX, and NeuN proteins. This is further evidence that 5-MTHF has a protective effect on hippocampal neurogenesis and neuronal survival.

5-MTHF transport from the blood into the CSF occurs mainly through the CP and then evacuates to the brain tissue, and cerebral folate transport is an essential component in CSF-CP signaling axes. Changes in CP polarity and loss of transport function, which are caused by Alzheimer’s disease and LOD, might give rise to the failure of folate transport across the CP, resulting in decreased folate levels in the CSF (Serot et al., 2001). In another study, it was found that the CP is involved in a variety of chronic stress-related diseases, and chronic stress might directly trigger changes in CP polarity and transport function (Sathyanesan et al., 2012). Tight junctions between highly polarized epithelial cells are the main structures that maintain the barrier and transport function of cells. ZO-1 is highly expressed in epithelial cells and endothelial cells and is crucial for tight junction stability and epithelial cell polarity (Hawkins and Davis, 2005). Notably, correct cell polarization is an unambiguous requirement for epithelial cells because polarity influences intracellular signal trafficking (Reinhold and Rittner, 2017). In this study, ZO-1 protein levels were significantly reduced in the CP of LOD rats, indicating disruption of tight junctions in the CP epithelium, which might hamper the expression of transporter proteins in the CP (Tsukita et al., 2001). The results of the *in vitro* experiments support this; CORT + D-gal conditions directly altered the CP cell polarity and reduced ZO-1 protein levels in primary CPECs, as well as decreasing the epithelial resistance. The *in vitro* and *in vivo* experiments demonstrated that luteolin effectively maintained CP tight junctions, upregulated ZO-1 protein levels in the CP, and increased CPEC resistance. Results consistent with our findings have been previously reported by others (Yuan et al., 2021); however, the exact mechanisms await further study.

Transport of substances by the CP epithelium is a polar transport, and this process is achieved by various transport proteins distributed in cell membranes. Disruption of the polarity of epithelial cells might affect the distribution of these transport proteins on the cell membrane, which in turn interferes with the transport of substances by epithelial cells (Tsukita et al., 2001; F et al., 2019; Yuan et al., 2021). Folate is anionic at physiological pH; therefore, it cannot spread across biological membranes through diffusion and must rely on specific transport systems to enter into cells and move epithelial cells (Alam et al., 2020b). In addition to being associated with the epithelial cell polarity, other pivotal factors in the 5-MTHF brain transport are the CP folate transport-related proteins, such as FRα, RFC, and PCFT (Molero-Luis et al., 2015; Pope et al., 2019). These transport proteins are expressed at specific locations in the CP and are required for the transport of 5-MTHF into the CSF (Zhao et al., 2017). FRα is embedded in the plasma membrane by a glycosylphosphatidylinositol anchor and mediates the uptake of folate into cells *via* receptor-mediated endocytosis at neutral to mildly acidic pHs (Grapp et al., 2013). PCFT, which has an optimum pH of 5.5, is expressed in the basolateral membrane of the CP and transports 5-MTHF from the basolateral membrane to the apical membrane of the CP where the RFC is located. Eventually, the RFC transports 5-MTHF into the CSF at the physiological pH (7.451) (Hou and Matherly, 2014). More importantly, FR-α binds 5-MTHF with high affinity to mediate the cellular uptake of folate. PCFT also binds 5-MTHF with high affinity at an acidic pH. The opposite is true for the RFC, meaning that the RFC has to work at a relatively high concentration of folic acid. The experimental results showed that FR-α was expressed similarly among the groups, which we considered to be related to the plasma 5-MTHF level in LOD rats and FR-α’s high affinity for folate. PCFT protein levels were significantly decreased in the CP of LOD rats, leading to a reduced level of 5-MTHF transport from the basolateral membrane to the apical membrane of the CP, which might also contribute to the decreased level of the RFC in LOD rats.

Among the correlations between the levels of FR-α, PCFT, RFC proteins, and CSF 5-MTHF, the correlation between the PCFT and the content of 5-MTHF in the CSF was the highest, indicating that the PCFT plays a critical role in delivering folate to the brain. The PCFT is a proton symporter that operates most efficiently at an acidic pH; the proton gradient across the cell membrane and the sodium-proton exchanger on the basolateral membrane provide the energy source for the uphill transport of folate into cells (Counillon and Pouyssegur, 2000; Serrano et al., 2012; Zhao et al., 2017). This demonstrated that the transport process of the PCFT is energy-consuming, and the downregulation of the PCFT level in the CP suggested that LOD rats might, to a certain extent, have abnormal energy metabolism, which also verifies the study by Linghu (Linghu et al., 2020) that chronic stress triggers impaired energy metabolism. A study also showed that aging might trigger impaired energy metabolism (Barzilai et al., 2012). The ATPase activity of the CP declines with age, especially decreases in the Na and K-ATPase activity (Kvitnitskaia-Ryzhova and Shkapenko, 1992). However, this was not the case in the present study. There was still a relatively high level of PCFT in the CP of the AGED rats. The PCFT is essential for intestinal folate absorption and transport into the central nervous system (Zhao et al., 2009). In addition, previous studies demonstrated that defects in the PCFT were observed in patients with hereditary folate malabsorption (Zhao et al., 2007). Even when blood folate levels in patients with hereditary folate malabsorption are recovered to, or exceed, the normal levels using folate supplementation, the CSF/blood folate ratio remains low. This is a convincing indication that in the presence of normal levels of plasma folate, the ability to transport 5-MTHF through the CP to the CSF is reduced because of a PCFT defect. Likewise, the results of this study showed that 5-MTHF levels were normal in the plasma of LOD rats, but a decrease was observed in the CSF. Stress affects cellular aging (Esch et al., 2018). The study also demonstrated that the use of proton pump inhibitors accelerated endothelial senescence and increased the risk of Parkinson’s disease and dementia (Yepuri et al., 2016; Lai et al., 2020; Rameau et al., 2021). We speculated that the LOD rats showed decreased levels of the PCFT, which might be related to dysfunctional or low-activity proton pumps. This speculation requires further experiment for confirmation.

Conversely, *in vitro* and *in vivo* assays showed that folate-related transporters in the CP were significantly upregulated by luteolin treatment, suggesting that luteolin could act directly on the CP to modulate folate transport protein levels. Consequently, we found that the PCFT and RFC are the targets of the anti-folate resistance pathway. According to the Herb database, luteolin appears to upregulate the expression of these proteins. This effect of luteolin to ameliorate 5-MTHF brain transport disorders suggested that targeting folate-related transporters holds a significant promise as a therapeutic option for diseases associated with folate brain transport disorders, such as neural tube defects, cerebral folate deficiency syndrome, and hereditary folate malabsorption, the effects of which, are currently having devastating effects on young children (Alam et al., 2020b).

Some limitations should be considered when interpreting our findings. How luteolin affects structural changes in the CP, how it alters the expression of folate transporter-related proteins, and what pharmacological targets are involved remain to be explored in depth. Alternatively, although the results of correlation analysis might suggest that the decrease in the folate content in the CSF and hippocampus is related to the reduction of the CP folate transporter expression to some extent, it is unclear whether the decreased folate content in the CSF and hippocampus is solely caused by the dysfunctional CP folate transporter; thus, the CP folate transporter knockout experiment should be used to address this issue.

## Conclusion

In summary, we noted that aging rats markedly exhibited depression-like behavior after exposure to CUMS, with cognitive dysfunction, mature hippocampal neuron loss, and neurogenesis impairment, reduced the 5-MTHF content in the CSF and the hippocampus, altered CP polarity, and decreased levels of folate transport-related proteins, which confirmed that LOD does involve impaired folate brain transport and that CSF with a reduced 5- MTHF content might trigger the hippocampal neurological dysfunction. This situation can be revered using luteolin, which improved the dysfunctional behavior triggered by the impaired folate transport and enhanced hippocampal neurogenesis. These findings indicated that the luteolin might have a great potential for the prevention and treatment of disorders related to folate brain transport disorders, including LOD.

## Data Availability

The original contributions presented in the study are included in the article/Supplementary Material, further inquiries can be directed to the corresponding authors.
